# Supplementary material for: Twin Study Provides Heritability Estimates for 2321 Plasma Proteins and Assesses Missing SNP Heritability
Source: J Proteome Res. 2025 May 27;24(6):2689–97. doi: 10.1021/acs.jproteome.4c00971 (PMC12150308; doi:10.1021/acs.jproteome.4c00971)
Supplement: Supplementary file 1 [file pr4c00971_si_001.pdf]

SUPPORTING INFORMATION

Twin study provides heritability estimates for 2,321 plasma proteins and assesses missing SNP heritability

Gabin Drouard[1] (*), Fiona A. Hagenbeek[1], Miina Ollikainen[1,2], Zhili Zheng[3], Xiaoling Wang[4], FinnGen[5], Samuli Ripatti[1,3,6], Matti Pirinen[1,6,7], Jaakko Kaprio[1] (*)

1. Institute for Molecular Medicine Finland (FIMM), HiLIFE, University of Helsinki, Helsinki 00014, Finland.
2. Minerva Foundation Institute for Medical Research, Helsinki 00290, Finland.
3. Broad Institute of MIT and Harvard, Cambridge, MA 02142, USA.
4. Georgia Prevention Institute, Medical College of Georgia, Augusta University, Augusta, GA 30912, USA
5. A complete list of the members of the FinnGen banner can be found in the Acknowledgements section.
6. Public Health, Faculty of Medicine, University of Helsinki, Helsinki 00014, Finland.
7. Department of Mathematics and Statistics, University of Helsinki, Helsinki 00014, Finland

(*) Corresponding authors: Gabin Drouard (gabin.drouard@helsinki.fi) & Jaakko Kaprio (jaakko.kaprio@helsinki.fi)

Table S1: Description of the proteins excluded from the analyses.

Table S2: Heritability estimates and influence of environmental effects on protein variability derived from classical twin models with twin correlations in monozygotic and dizygotic twin pairs.

Table S3: Twin-based heritability estimates next to SNP-based heritability estimates from Sun et al. (2023) for overlapping proteins.
